# Geochemical markers and polycyclic aromatic hydrocarbons in solvent extracts from diesel engine particulate matter

**DOI:** 10.1007/s11356-015-5996-z

**Published:** 2016-01-04

**Authors:** Monika Fabiańska, Barbara Kozielska, Piotr Bielaczyc, Joseph Woodburn, Jan Konieczyński

**Affiliations:** Faculty of Earth Sciences, University of Silesia, 60 Bedzinska St, 41-200 Sosnowiec, Poland; Faculty of Power and Environmental Engineering, Silesian University of Technology, 22B Konarskiego St, 44-100 Gliwice, Poland; BOSMAL Automotive Research and Development Institute Ltd, 93 Sarni Stok St, 43-300 Bielsko-Biala, Poland; Institute of Environmental Engineering, Polish Academy of Sciences, 34 M Sklodowskiej-Curie St, 41-819 Zabrze, Poland

**Keywords:** PAH, GC-MS, Diagnostic ratios, Geochemical ratios, CI

## Abstract

Exhaust particulate from compression ignition (CI) engines running on engine and chassis dynamometers was studied. Particulate dichloromethane extracts were qualitatively and quantitatively analyzed for polycyclic aromatic hydrocarbons (PAHs) and biomarkers by gas chromatography with flame ionization detector (GC-FID) and gas chromatography-mass spectrometry (GC-MS). PAH group profiles were made and the PAH group shares according to the number of rings (2 or 3; 4; 5 or more) as well as diagnostic indices were calculated. Values of geochemical ratios of selected biomarkers and alkyl aromatic hydrocarbons were compared with literature values. A geochemical interpretation was carried out using these values and biomarker and alkyl aromatic hydrocarbon distributions. It has been shown that geochemical features are unequivocally connected to the emission of fossil fuels and biofuels burned in CI engines. The effect of the exothermic combustion process is limited to low-molecular-weight compounds, which shows that the applied methodology permits source identification of PAHs coexisting in the particulate emitted.

## Introduction

One of the main sources of polycyclic aromatic hydrocarbons (PAHs) emission are fuels used in automotive engines, particularly lower-power compression ignition (CI) engines (Marr et al. 1999). Small diesel engines, working in machines other than road vehicles, emit a disproportionate amount of primary solid and gas pollutants per unit of fuel consumed (by mass). These contaminants are precursors of secondary pollutants occurring in the air, i.e., volatile organic compounds, particulate matter (PM), and organic carbon (Gordon et al. 2013).

It has been shown that the formation of secondary air pollutants (secondary organic aerosol—SOA) is affected by the number of carbon atoms and aromatic compounds in the fuel used. When fuels richer in heavier hydrocarbons and aromatic compounds are combusted, more SOA is formed (Jathar et al. 2013). The use of particulate filters for purifying the exhaust gas of CI engines leads to a reduction in emissions of genotoxic substances. The degree of elimination of these emissions depends on the type and on the filter’s oxidation ability (Heeb et al. 2010).

Specific risks to human health occur as a result of the PAHs present in particulates emitted by vehicles featuring CI engines. Diesel particulate has a large specific surface area, onto which may adsorb various toxic, mutagenic, and carcinogenic compounds (e.g., PAHs, nitro-PAHs, oxy-PAHs). Health hazards comprise lung cancer, allergic reactions, asthma, etc. (Kumagai and Taguchi 2007). However, current knowledge on the share of specific individual PAHs is insufficient. New possibilities were provided by testing for coexisting PAHs and biomarkers, geochemical substances characterized by their persistence, in exhaust gas. Distributions of hopanes, steranes, and PAHs in the fractions of PM emitted from heavy-duty diesel vehicles and the composition of PAHs depend on the operating conditions and the type of engine (Riddle et al. 2007). 5α-Cholestane, *n*-hexacosane, *n*-triacontane, 1,2-benzo[a]anthracene, and coronene were considered semi-volatile under atmospheric conditions (May et al. 2012). It has been found that chemical transformations in the air and the movement of air cause the utility molecular diagnosis ratios (MDRs) to indicate that the source of PAH emissions is limited (Katsoyiannis et al. 2011).

The aims of the study presented in this paper were to find whether:

- Occurrences and distributions of organic compounds adsorbed on diesel-emitted particulate (DEP) preserve the geochemical features of the source fossil fuel and bio-fuel additives;
- There are correlations between geochemical and PAHs diagnostic ratios enabling determination of the share of emission sources to the PAH concentration in ambient air.

## Material and method

### Test vehicles

To obtain the DEP samples, passenger cars, commercial vehicles, marine, and bus engines were used (Table 1) which represent a broad range of engine and aftertreatment technology. Specifically, both engines with and without diesel particulate filters (DPFs) were included in the study.

**Table 1:** Basic details of the test vehicles, PM sampling, particulate sample numbers, and DEP extract yields (% wt)

Vehicle/engine number	Vehicle/engine type	Approx. displacement (dm^3^)	Tested on blend	Aftertreatment system	Test cycle	Sample number	DEP extract yield (% wt)
1	PC	1.3	B5	DOC; no DPF	UDC	1	27.3
					EUDC	2	26.4
2	LCV	3.0	B5	DOC + DPF	UDC	3	26.3
					EUDC	4	25.6
3	LCV	2.8	B5	DOC; no DPF	UDC+EUDC	5	28.3
4	Marine recreational engine	2.0	B7	None	EPA 1042	6	35.6
5	PC	1.3	B5	DOC + DPF	UDC	7, 11	24.1; 24.8
					EUDC	8, 12	23.4; 22.5
					UDC	9, 13	23.9; 24.2
					EUDC	10, 14	21.9; 22.8
					UDC+EUDC	15, 16, 17	27.5; 25.6; 26.0
6	PC	1.9	B5	DOC; no DPF	UDC+EUDC	18	33.9
7	Bus	7.7	B5	DOC; no DPF	ESC 4-7	19	18.2

*PC* passenger car, *B5/B7* diesel blend with 5 %/7 % (vol.) fatty acid methyl ester content, *DOC* diesel oxidation catalyst, *UDC* urban driving cycle, *EUDC* extra-urban driving cycle, *LCV* light commercial vehicle, *DPF* diesel particulate filter, *EPA* US Environmental Protection Agency, *ESC* European Stationary Cycle

### Sampling of DEP and its preparation

DEP samples were collected on Whatman Grade QMA in tests conducted in the exhaust emissions laboratory at BOSMAL Automotive Research and Development Institute (Poland) The BOSMAL Automotive Research and Development Institute (Poland) is climate-controlled test facility which meets all the demands of the latest EU and US automotive regulations; in this study, tests were carried out according to the EU test procedure. The basic approach requires that the test vehicles be tested on a chassis dynamometer (AVL Zöllner 48” compact, single-roll) and driven over the EU test cycle—the New European Driving Cycle (NEDC). This well-known and widely used driving cycle commences from a cold start (at 20–30 °C) and consists of two phases: the initial cold start urban phase (urban driving cycle—UDC), followed by the extra-urban driving cycle (EUDC). These two cycles make up the NEDC as a whole, but results from the two phases can be interpreted separately, to cover different driving conditions (urban cold start and extra-urban hot running). The legislative approach requires that exhaust gas is conveyed to a dilution tunnel, from which samples of gaseous pollutants are taken for analysis (not the focus of this paper). A particulate sampling system connected to the dilution tunnel draws a portion of the exhaust gas through a filter assembly. The laboratory’s particulate sampling system is fully compliant with EU and US legislation. The filters used were Whatman Grade QMA, of diameter 4.7 cm. Flow rates were adjusted according to the vehicle under test, in line with good automotive testing practice. Samples obtained from engines were collected in a similar manner, but with the engine alone running on an engine (as opposed to chassis) dynamometer. In this case, an eddy-current dynamometer absorbs the engine’s power output to simulate load. The exhaust extraction system contains a particulate sampling system (Whatman Grade QMA filters, of diameter 7.0 cm). Embedded into the test stand are a number of sensors and dedicated emissions analyzers (Horiba) and a smoke meter (AVL), all of which monitor the conditions and engine operation to ensure repeatability.

Further, detailed information on this laboratory, its climatic chamber, its range of emissions testing equipment, and its chassis dynamometer is presented elsewhere (Bielaczyc et al. 2011).

Diesel particulate was extracted with dichloromethane (DCM) in an ultrasonic bath. Each extract was divided into two parts: one for investigation of biomarkers on gas chromatography-mass spectrometry (GC-MS) and the second one for PAHs via gas chromatography with flame ionization detector (GC-FID). That one was percolated, washed, and He-dried. Dry residue was diluted in propanol-2 and distilled water was added to a volumetric alcohol/water ratio of 15/85. For selective purification, the samples were solidified (SPE) via extraction on C-18 columns (Supelclear^TM^ ENVI-18 Tubes, Supelco USA). PAHs were eluted with DCM. The PAHs extracts were worked up to a volume of 0.5 cm^3^ using He.

### Gas chromatography of DEP extracts

The SPE extracts were analyzed using a Clarus 500 Perkin Elmer gas chromatograph equipped with a Restek RTX-5 capillary column (30 m × 0.32 mm × 0.25 μm film thickness) and FID detector. Sixteen PAHs were analyzed: naphthalene (Na), acenaphtene (Acy), acenaphthylene (Ace), fluorene (F), phenanthrene (Ph), anthracene (An), fluoranthene (Fl), pyrene (Pyr), benzo[a]anthracene (BaA), chrysene (Ch), benzo[b]fluoranthene (BbF), benzo[k]fluoranthene (BkF), benzo[a]pyrene (BaP), dibenzo[a,h]antracene (DBA), benzo[g,h,i]perylene (BghiP), and indeno[1,2,3-cd]pyrene (IP). The temperature program began at 60 °C, held for 4 min, then the temperature was increased by 10 °C min^−1^ up to 280 °C, held for 14 min. The carrier gas (He) flow rate was 1.5 cm^3^ min^−1^. The evaporator temperature was 240 °C and the detector temperature was 280 °C.

The quantitative analysis was done based on the calibration curves for 16 standard PAHs. The linear correlation between the peak surface areas and the PAH concentrations was checked within the range 10–40 ng μl^−1^ (correlation coefficient 0.99, PAH Mix PM-611 Ultra Scientific standard of concentration 100 μg ml^−1^ of each PAH was used). The analysis of each sample-series was accompanied by the analysis of a blank sample. It consisted in the application of the whole analytical procedure to a clean quartz fiber filter. The blank result was used to adjust the PAH concentration, but only if the blank exceeded 10 % of the PAH concentration.

The apparatus, details of PM extraction, and the analysis parameters were described in the studies (Rogula-Kozłowska et al. 2013; Kozielska et al. 2015).

### Gas chromatography-mass spectrometry of DEP extracts

Prior to GC-MS analyses for biomarker content, the extracts were not separated into compound groups, due to the low extractability. An Agilent 6890 gas chromatograph with a DB-35 column (60 m × 0.25 mm id, 0.25 μm stationary phase film) coupled with an Agilent Technology 5973 mass spectrometer was used. The experimental conditions were as follows: carrier gas—He; temperature—50 °C (isothermal for 2 min); heating rate—up to 175 °C at 10 °C min^−1^, to 225 °C at 6 °C min^−1^, and finally, to 300 °C at 4 °C min^−1^. The final temperature (300 °C) was held for 20 min. The mass spectrometer was operated in the ionisation mode (70 eV, full scan) and scanned from 50 to 650 Da.

The compounds were identified by their mass spectra, comparison of peak retention times with those of standard compounds, and literature (Philp 1985; Wiley/NBS Registry of Mass Spectral Data 2000). Geochemical parameters were calculated using peak areas acquired in manual integration mode.

## Results and discussion

Diesel particles contain elementary carbon, organic carbon, and small amounts of sulfate, nitrate, trace elements, water, and unidentified components (US EPA 2002). PM sampled from diesel exhaust is rich in organic matter. The extracts contained on average 26.3 % (18.2–33.9 %) organic matter by mass. The US EPA (2002) reported that in diesel particles (PM_2.5_) are on average 19 % organic carbon (7–49 %).

### PAHs occurrence and distributions in DEP extracts

DEP mainly contains two, three-, and four-ring PAHs, whereas gasoline exhaust particulate can contain significant amounts of five- and six-ring PAHs (Miguel et al. 1998). The main components of particulate matter from the diesel exhaust investigated here were lighter PAHs: mainly F, Ph, and Pyr, as found in previous studies (Miguel et al. 1998; Wingfors et al. 2001; Kendall et al. 2002). Khalili et al. (1995) showed that two- and three-ring PAHs comprise about 76 % (wt) of the total PAHs sampled from diesel exhaust. In this investigation, it was found when analyzing PM emitted from typical diesel engines powered by B5 that in 11 samples a sum of two, three-, and five-ring PAHs significantly exceeded 60 % (wt) (Table 2). Only in three samples did the percentage content of five- and six-ring PAHs reach 40 % (wt) of the total PAHs or exceeded this value. In the case of the marine recreational engine, the two- and three-ring PAHs relative content was 98 %. This confirms the results of the authors mentioned above. Over 50 % (wt) of five- and six-ring PAHs contents were found only for the modern common rail automotive engine and the old bus engine.

**Table 2:** Mass shares of PAHs groups considering number of rings in the particle (wt)

Sample number	Number of rings in the molecule		
	2–3	4	5–6
1	9.20	51.40	39.40
2	86.56	13.44	-
3	79.56	18.31	2.14
4	82.36	15.55	2.09
5	83.27	14.76	1.97
6	98.14	1.55	0.31
7	3.47	95.47	1.06
8	5.38	90.69	3.93
9	15.44	73.69	10.87
10	41.70	44.08	14.23
11	7.79	71.72	20.49
12	33.86	8.21	57.93
13	36.85	45.75	17.41
14	68.89	27.36	3.76
15	53.53	43.34	3.13
16	46.74	29.98	23.28
17	27.56	25.44	47.00
18	11.48	36.78	51.74
19	12.26	30.41	57.33

Value ranges of diagnostic ratios (DRs) of PAHs are shown in Table 3. In a limited way, they can be used to identify source(s) of PM emission, but alone they are insufficient. For the diesel PM investigated here, the following DRs were applied: Ph/(Ph+An), Fl/(Fl+Pyr), BaP/(BaP+Ch), BaA/(BaA+Ch). Their values are very close to those calculated by other researchers (Rogge et al. 1993; Khalili et al. 1995; Yang et al. 1998; Kavouras et al. 1999; Alves et al. 2001; Kavouras et al. 2001; Mandalakis et al. 2002; Fang et al. 2004; Ravindra et al. 2006). Additionally, F/(F+Pyr) was calculated as being 0.63 and 0.31 for diesel petrofuel and biofuel, respectively. Average values of An/(An+Ph) (0.34—diesel petrofuel; 0.38—biofuel) are lower than those found by Kuo et al. (2013) for diesel-dominated routes. Values of the Fl/Pyr ratio for both fuel types were similar, while for both fuels Ph/An and Pyr/BaP values differed significantly.

**Table 3:** Average values of selected diagnostic (DRs) ratios for DEP

Diagnostic ratio	Value	References
Ph/(Ph+An)	0.65 (0.42–0.90)	This study^a^
	0.51 (0.30–0.72)	This study^b^
	0.75	Kavouras et al. (2001)
	0.65	Alves et al. (2001)
F/(F+Pyr)	0.63 (0.30–0.94)	This study^a^
	0.31 (0.13–0.40)	This study^b^
An/(An+Ph)	0.34 (0.10–0.51)	This study^a^
	0.38 (0.28–0.47)	This study^b^
	0.43	Kuo et al. (2013)
Fl/(Fl+Pyr)	0.56 (0.23–0.69)	This study^a^
	0.63 (0.57–0.66)	This study^b^
	>0.5	Rogge et al. (1993); Mandalakis et al. (2002); Fang et al. (2004)
	0.60–0.70	Kavouras et al. (2001)
	0.41	Kavouras et al. (2001); Kuo et al. (2013)
Fl/Pyr	1.73 (1.24–2.28)	This study^a^
	1.74 (1.31–1.96)	This study^b^
Ph/An	1.83 (1.32–2.32)	This study^a^
	1.73 (1.11–2.61)	This study^b^
BaP/(BaP+Ch)	0.47 (0.22–0.86)	This study^a^
	0.61 (0.33–0.79)	This study^b^
	0.68	Khalili et al. (1995)
	0.5	Ravindra et al. (2006)
BaA/BaP	0.75 (0.45–1.17	This study^a^
	0.96 (0.80–1.21)	This study^b^
	1.0	Li and Kamens (1993)
Pyr/BaP	0.66 (0.28–1.11)	This study^a^
	0.42 (0.32–0.75)	This study^b^
BaA/(BaA+Ch)	0.73 (0.62–0.95)	This study^a^
	0.61 (0.32–0.75)	This study^b^
	0.38–0.64	Kavouras et al. (2001)
	0.23–0.89	Yang et al. (1998)
	0.22–0.55	Simcik et al. (1999)
	0.30	Kuo et al. (2013)
IP/(IP+BghiP)	0.35–0.70	Kavouras et al. (1999); Ravindra et al. (2006)
BbF/BkF	>0.5	Pandey et al. (1999); Park et al. (2002)
	2.32	Ravindra et al. (2008)
IP/BghiP	>1	Caricchia et al. (1999)
	1	Kavouras et al. (1999)
Pyr/BaP	>10	Oda et al. (2001)

^a^Only petrogenic compounds found

^b^Petrogenic and biogenic compounds present

### Geochemical features of DEP extracts

Geochemical compounds present in the DEP extracts included the following:

- *n*-Alkanes (*m/z* = 71), mostly in the range of *n*-C_16_–*n*-C_33_, with accompanying branched alkanes and long-chain alkyl cyclohexanes;
- Acyclic isoprenoids (*m/z* = 71, 183): pristane and phytane; in some extracts norpristane (a pristane degradation product);
- Steranes (*m/z* = 217) from C_27_ (cholestanes) to C_29_ (stigmastanes), often accompanied by diasteranes (*m/z* = 259);
- Pentacyclic triterpenoids (*m/z* = 191);
- Aliphatic derivatives of light aromatic hydrocarbons (naphthalene, phenanthrene, and biphenyl), together with alkyl chrysenes and alkyl pyrenes (in some samples).

These compounds, originating from fossil fuels, were accompanied by polar compounds such as methyl esters of fatty acids, probably from biofuel additives. The most common were methyl ester of octadecenoic acid and methyl ester of hexadecenoic acid, which occurred in higher concentrations, but methyl esters of pentadecenoic, nonadecenoic, and dodecenoic acids were also identified. The other compounds that could be ascribed to biofuels include acyclic isoprenoids like farnesol (3,7,11-trimethyl-2,6,10-dodecatrien-1-ol) and squalene, since they occur in recent plants.

Generally, PM from combustion without an aftertreatment system is the richest in geochemical compounds (i.e., samples 5 and 6). The increase in the number of biomarkers identified is also clearly visible when comparing samples 1 and 2, and 3 and 4 from the same vehicle operating in the UDC and EUDC test cycles, with particulate matter from the EUDC containing higher concentrations of organic compounds and better distributions, with well identified compounds.

To characterize the origin(s) of organic compounds in DEP, geochemical ratios and indices were applied (Tables 4 and 5).

**Table 4:** Values of geochemical ratios of biomarkers found in DEP extracts

Sample number	CPI_17-22_	CPI_24-34_	Σ2/Σ1	*n*-C_23_/*n*-C_31_	Pr/Ph	Pr/*n*-C_17_	Ph/*n*-C_18_	(Pr+Ph)/(*n*-C_17_+*n*-C_18_)	Ts/(Ts+Tm)	C_31_S/(S+R)	C_29_Ts/(C_29_+C_29_Ts)	βα/(αβ+βα)	C_29_ 20S/(20S+20R)	C_29_αββ/(ααα+αββ)	C_27_ααα/C_29_ααα
	(1)	(2)	(3)	(4)	(5)	(6)	(7)	(8)	(9)	(10)	(11)	(12)	(13)	(14)	(15)
1^a^	1.13	0.96	3.73	–	1.26	0.30	0.36	0.32	0.49	0.57	0.15	0.17	0.60	0.53	0.82
2^a^	0.59	1.18	1.42	1.59	1.24	0.78	0.60	0.69	–	–	–	–	–	–	–
3^a^	1.28	1.22	5.09	1.29	5.70	2.17	0.42	1.23	–	0.63	–	–	0.63	0.59	3.39
4^a^	1.15	1.17	4.12	3.15	6.36	2.60	0.56	1.74	–	0.62	–	–	–	–	–
5^a^	2.28	1.22	3.87	5.85	3.06	1.26	0.40	0.83	0.41	0.59	0.14	0.06	0.50	0.49	1.01
6^a^	1.08	0.96	1.82	4.29	3.70	0.91	0.40	0.72	0.45	0.59	0.18	0.08	0.55	0.54	0.66
7^b^	1.29	7.11	2.43	–	–	–	–	–	–	–	–	–	–	–	–
8^b^	1.39	5.67	0.99	–	–	0.11	–	0.11	–	–	–	–	–	–	–
9^b^	0.27	0.63	0.91	2.77	0.52	0.69	0.25	0.32	–	–	–	–	–	–	–
10^b^	0.37	0.98	0.71	0.55	0.88	0.96	0.49	0.64	–	–	–	–	–	–	–
11^b^	1.57	4.96	2.97	1.41	0.73	0.80	0.63	0.69	–	0.56	–	––	–	–	–
12^**b**^	1.05	4.94	1.23	–	–	–	–	–	–	–	–	–	–	–	–
13^b^	0.69	0.48	1.49	2.95	0.76	1.06	1.26	1.16	–	0.53	–	0.21	–	–	–
14^b^	0.50	0.28	0.99	1.14	0.52	1.27	1.37	1.34	–	0.58	–	0.29	–	–	–
15^a^	1.07	–	0.27	–	0.25	1.00	2.33	1.93	0.58	–	–	–	–	–	–
16^a^	1.24	0.90	1.61	–	0.55	0.89	1.68	1.28	–	–	–	–	–	–	–
17^a^	0.94	1.37	1.69	1.29	0.43	1.29	1.05	1.12	0.65	–	–	–	–	–	–
18^b^	0.64	1.10	0.70	3.57	2.87	1.23	0.20	0.43	0.54	0.57	–	0.17	0.27	0.52	0.79
19^a^	0.94	1.32	0.08	–	1.04	0.36	0.31	0.33	0.44	0.59	0.14	–	–	–	–

(1) CPI_17-23_ = 0.5 [(*n*-C_17_+*n*-C_19_+*n*-C_21_)/(*n*-C_18_+*n*-C_20_+*n*-C_22_)] + [(*n*-C_19_+*n*-C_21_+*n*-C_23_)/(*n*-C_18_+*n*-C_20_+*n*-C_22_)]; Carbon Preference Index; *m/z* = 71; Kotarba and Clayton 2003. (2) CPI_24-34_ = 0.5{[(*n*-C_25_+*n*-C_27_+*n*-C_29_+*n*-C_31_+*n*-C_33_) /( *n*-C_24_+*n*-C_26_+*n*-C_28_+*n*-C_30_+*n*-C_32_)] + [(*n*-C_25_+*n*-C_27_+*n*-C_29_+*n*-C_31_+*n*-C_33_) / (*n*-C_26_+*n*-C_28_+*n*-C_30_+*n*-C_32_+*n*-C_34_)]; Carbon Preference Index; *m/z* = 71; thermal maturity (Bray and Evans 1961). (3) Σ2/Σ1 = [Σ (from *n*-C_23_ to *n*-C_37_)]/[Σ (from *n*-C_11_ to *n*-C_22_) ]; *m/z* = 71, source indicator (Tissot and Welte 1984). (4) *n*-C_23_/*n*-C_31_; *m/z* = 71 source indicator (Pancost et al. 2002). (5) Pr/Ph = pristane/phytane; parameter of environment oxicity (with exception of coals); *m/z* = 71 (Didyk et al. 1978). (6) Pr/*n*-C_17_ = pristane/*n*-heptadecane; *m/z* = 71 (Leythaeuser and Schwartzkopf 1985). (7) Ph/*n*-C_18_ = phytane/*n*-octadecane; *m/z* = 71 (Leythaeuser and Schwartzkopf 1985). (8) (Pr+Ph)/(*n*-C_17_+ *n*-C_18_)=(pristane+phytane)/(*n*-heptadecane+n-octadecane); *m/z* = 71 (Leythaeuser and Schwartzkopf 1985). (9) C_31_S/(S+R) = 17α(H),21β(H)-29-homohopane 22S/(17α(H),21β(H)-29-homohopane 22S+17α(H),21β(H)-29-homohopane 22R); *m/z* = 191; thermal maturity parameter (Peters et al. 2005). (10) Ts/Ts+Tm = 18α(H)-22,29,30-trisnorneohopane/(18α(H)-22,29,30-trisnorneohopane+17α(H)-22,29,30-trisnorhopane); *m/z* = 191; thermal maturity parameter (Peters et al. 2005). (11) C_30_βα/(αβ+βα) = 17β(H),21α(H)-29-hopane C_30_/(17α(H),21β(H)-29-hopane C_30_+17β(H),21α(H)-29-hopane C_30_); *m/z* = 191, (Seifert and Moldowan 1986). (12) C_30_ββ/ββ + αβ+βα = 17β(H),21β(H)-29-hopane C_30_/(17β(H),21β(H)-29-hopane C_30_+17β(H),21α(H)-29-hopane C_30_); *m/z* = 191, (Seifert and Moldowan 1986). (13) C_29_αααS/(S+R) = a ratio of C_29_-5α,14α,17α(H)-stigmastane 20S to a sum of its diastereomers 20S and 20R; *m/z* = 217, Seifert and Moldowan 1986). (14) C_29_ααα/(C_29_ααα + C_29_αββ) = the ratio of C_29_-5α,14α,17α (H)-stigmastane (20S + 20R) to the sum of its diastereomers C_29_-5α,14α,17α(H)-stigmastane (20S+20R)+C_29_-5α,14β,17β(H)-stigmastane (20S+20R), *m/z* = 217 (Seifert and Moldowan 1986). (15) C_27_ααα/C_29_ααα = the ratio of the sum of cholestane to the sum of stigmastane diastereomers (Peters et al. 2005). “–” compounds not detected; or compounds detected but concentrations too low to calculate a parameter value

^a^Only petrogenic compounds detected

^b^Petrogenic and biogenic compounds present

**Table 5:** Values of geochemical ratios of alkyl-based aromatic hydrocarbons found in DEP extracts

Sample number	MNR	DNR-1	TNR-1	TNR-2	TNR-5	MPI-3	MPI-1	DMPR	MPyR	MChR	(3-MB+4MB)/ DBF	R_c_[%]
	(1)	(2)	(3)	(4)	(5)	(6)	(7)	(8)	(9)	(10)	(11)	(12)
1^a^	–	–	–	–	–	1.00	0.53	0.51	0.54	0.14	–	0.72
2^a^	–	–	–	–	–	1.40	0.55	0.26	–	–	–	0.73
3^a^	–	–	–	–	–	0.92	0.15	–	0.75	0.11	–	0.49
4^a^	–	–	–	–	–	–	–	–	–	–	–	–
5^a^	–	–	–	–	–	–	0.44	0.42	0.65	0.13	–	0.66
6^a^	–	–	–	–	–	1.20	0.71	0.48	0.64	0.26	–	0.82
7^b^	–	–	–	–	–	–	–	–	–	–	–	–
8^b^	–	–	–	–	–	–	–	–	–	–	–	–
9^b^	–	–	–	–	–	1.29	0.90	–	–	–	–	0.94
10^b^	–	–	–	–	–	1.34	0.61	–	–	–	–	0.77
11^b^	–	–	–	–	–	1.79	0.12	–	–	–	–	0.51
12^b^	–	–	–	–	–	–	–	–	–	–	–	–
13^b^	–	–	–	–	–	0.94	0.19	0.97	0.58	–	0.02	0.51
14^b^	–	–	–	–	–	1.48	0.29	–	–	–	0.04	0.57
15^a^	1.33	–	0.86	0.81	0.46	1.48	0.13	–	0.49	–	0.15	0.48
16^a^	–	–	–	–	–	3.42	0.79	–	–	–	0.24	0.87
17^a^	0.68	–	0.46	0.45	0.43	–	0.52	–	–	–	0.28	0.71
18^b^	–	–	–	–	–	1.61	0.25	–	–	–	0.13	0.55
19^a^	1.67	9.97	0.72	0.76	0.30	1.55	0.39	0.33	0.61	–	0.54	0.64

(1) MNR = 2-methylnaphthalene/1-methylnaphthalene; *m/z* = 142; thermal maturity (Radke 1988). (2) DNR-1 = (2,6-dimethylnaphthalene+2,7-dimethylnaphthalene)/1,5-dimethylnaphthalene; *m/z* = 156, thermal maturity (Radke 1988). (3) TNR-1 = (1,3,7-trimethylnaphthalene+2,3,6-trimethylnaphthalene)/(1,3,5-trimethylnaphthalene+1,4,6-trimethylnaphthalene+1,3,6-trimethylnaphthalene); *m/z* = 170, thermal maturity (Radke 1988). (4) TNR-2 = (1,3,7-trimethylnaphthalene+2,3,6-trimethylnaphthalene)/(1,3,5-trimethylnaphthalene+1,4,6-trimethylnaphthalene+1,3,6-trimethylnaphthalene); *m/z* = 170*,* thermal maturity (Radke 1988). (5) TNR-5 = 1,2,5-trimethylnaphthalene/(1,2,5-trimethylnaphthalene+1,2,7-trimethylnaphthalene+1,6,4-trimethylnaphthalene); *m/z* = 170, source (Radke 1988). (6) MPI-3 = (2-methylphenanthrene+3-methylphenathrene)/(1-methylphenathrene+9-methylphenathrene); *m/z* = 192; thermal maturity (Radke 1988). (7) MPI-1 = 1.5(2-methylphenanthrene+3- methylphenanthrene)/(phenanthrene+1-methylphenanthrene+9-methylphenanthrene); thermal maturity (Radke 1988). (8) DMPR = dimethylphenanthrene ratio ([3,5-+2,6-+2,7-DMP]/[1,3-+3,9-+2,10-+3,10-+1,6-+2,9-+2,5-DMP]), *m/z* = 206; thermal maturity (Radke 1988). (9) MPyR = 2-methylpyrene/(1-methylpyrene+2-methylpyrene); *m*/*z* = 216, thermal maturity parameter (Kruge 2000). (10) MCHR = 2-methylchrysene/(methylbenzoanthracenes+2-methylchrysene+6-methylchrysene+1-methylchrysene), *m*/*z* = 242, thermal maturity (Kruge 2000). (11) (3-MB+MB)/DBF = (3-methylbiphenyl+4-methylbiphenyl)/dibenzofurane; *m/z* = 168; thermal maturity parameter (Radke et al. 2000). (12) calculated vitrinite reflectance; R_c_ = 0.60 MPI-1+0.40 (Radke 1988). “–” compounds not detected; or compounds detected but concentrations too low to calculate a parameter value

^a^Only petrogenic compounds detected

^b^Petrogenic and biogenic compounds present

#### *n*-Alkanes

**Fig. 1:**
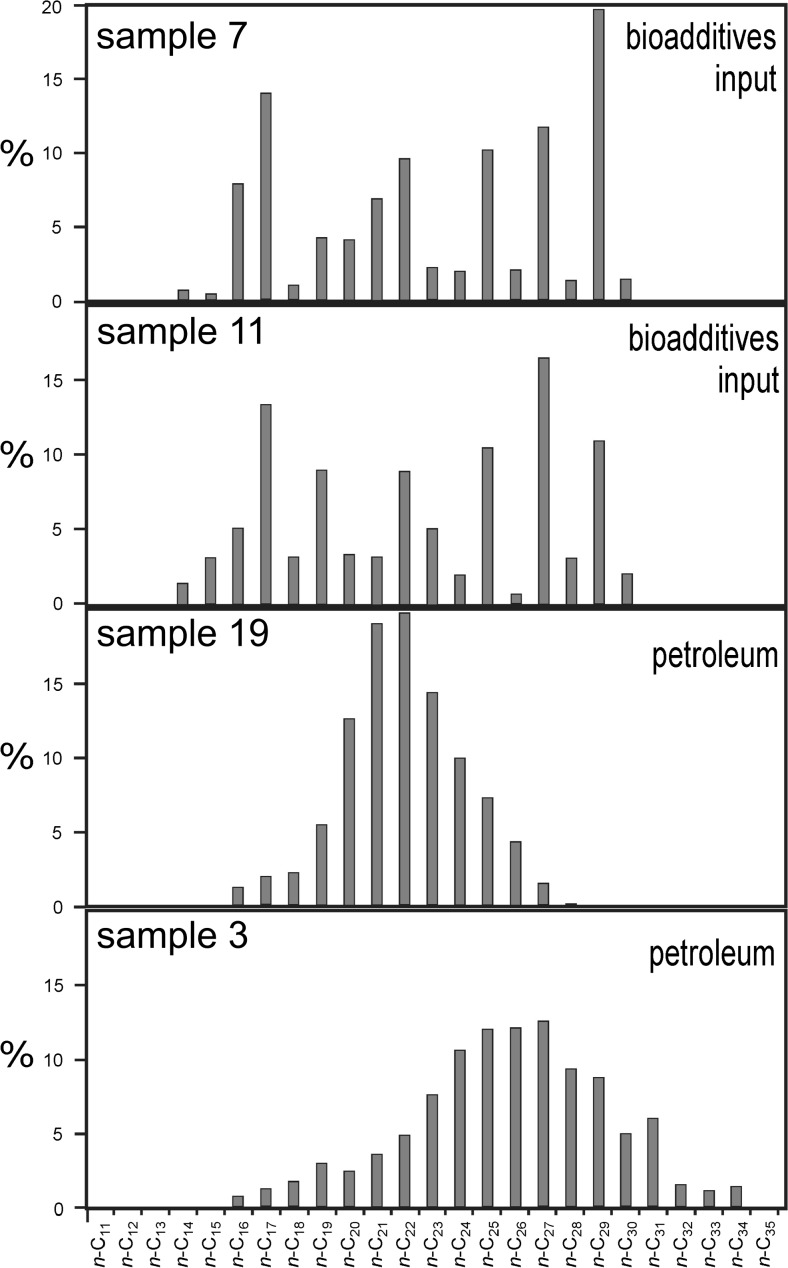
Relative percentage distributions of *n*-alkanes occurring in DEP extracts (based on *m/z* = 71 ion chromatograms); samples: a, 7; b, 11; c, 19; d, 3

*n*-Alkane profiles seem to be most diagnostic for the origin(s) of organic compounds present in DEP. In the extracts investigated, they occurred in the range from *n*-C_12_ (most from *n-*C_17_) to *n-*C_34_, with three different distribution types related to the origin of these compounds (Fig. 1):

- Monomodal with a *n*-C_23_–*n*-C_25_ maximum, a smooth *n*-alkanes outline, and both CPI_17-23_ and CPI_24-34_ close to 1.0 (samples: 1, 3-6, 15-17, and 19); petrochemical origin;
- Bimodal with maxima for *n*-C_16_–*n*-C_17_ and *n*-C_29_ with high CPI_24-34_ caused by the input of *n*-alkanes from bioadditives (plant-derived oil) to the fuel (samples: 8, 9, 11, 12);
- Variable, but with CPI_17-23_ much lower than 1.0 and low values of CPI_24-34_ (samples: 2, 9, 10, 13, 14, and 18), as the possible source fatty alcohols can be suggested or fatty acids whose decarboxylation can produce such *n*-alkane distributions since *n*-C_16_, *n*-C_18_, and/or *n*-C_14_ prevail in most vegetation, e.g., in rapeseed or sunflower oils (e.g., Simpson et al. 1999; Radke et al. 2000).

**Fig. 2:**
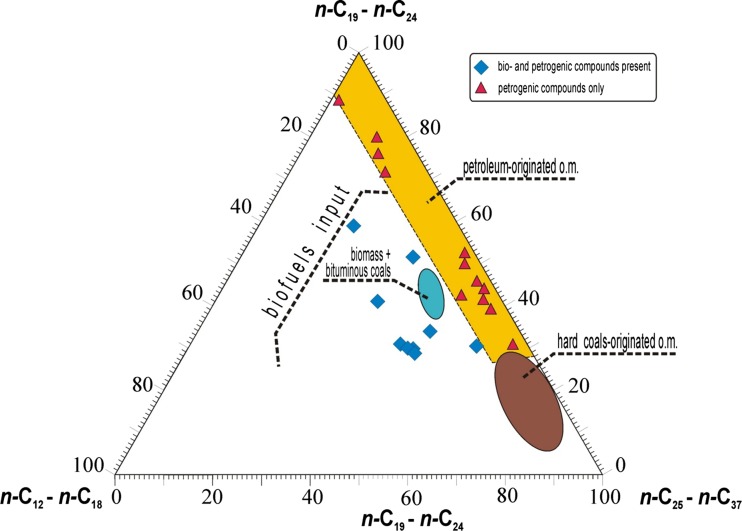
Ternary plot of *n*-alkanes showing relative percentage contents of *n*-C_11_–*n*-C_18_, *n*-C_19_–*n*-C_24_, and *n*-C_25_–*n*-C_38_ *n*-alkanes in DEP extracts

The relative percentage composition of three groups of *n*-alkanes plotted in a ternary diagram (Fig. 2) reveals their possible origin in relation to other DEP emitted from bioadditives and fossil fuels investigated by other authors (Knothe et al. 2006; Kanya et al. 2007).

All samples containing only petrochemical compounds are poor in short-chain *n*-alkanes (*n*-C_12_-*n*-C_18_), with variable content of the other two groups. It is also reflected by low Σ2/Σ1 values (Table 4). It is worth pointing out that these samples do not overlap with the bituminous coal region, which can help in future differentiation of contamination sources. Compared to fossil fuels, there is a significant increase of short chain *n*-alkanes relative contents in samples which contain preserved biogenic compounds. The same was true for biomass co-combusted with bituminous coals. This may indicate that co-combustion favors preservation of lighter compounds or be related to the *n*-alkanes profile in planted-derived oil richer in short chain compounds.

**Fig. 3:**
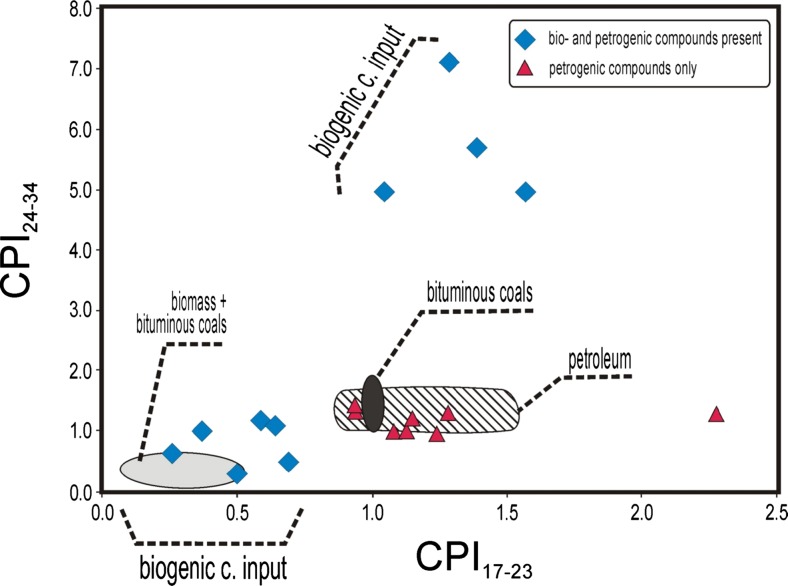
CPI_17-23_ versus CPI_24-24_ diagram showing the dependence on the fuel type

To differentiate distribution types, a CPI_17-23_ versus CPI_24-34_ plot was applied (Fig. 3). Input of fatty alcohols and fatty acids caused a shift of samples into a region of low values of both indices, i.e., a predominance of even-carbon-number *n*-alkanes. The same trend was found for particulate from co-combustion of biomass and bituminous coals. Since a feature is unusual in geochemical samples and was found only in uncommon immature crude oils, usually deriving from organic matter deposited in hypersaline carbonate environments where aerobic and anaerobic bacteria have degraded the remains of blue-green algae (Dembicki et al. 1976; Jiamo et al. 1990), we propose using it as an indicator of biofuel additives in DEP. However, the high values CPI_24-34_ could also be related to vegetable oil sources, containing fatty acids with predominantly even carbon atom numbers in the molecule. After decarboxylation, an *n*-alkane distribution with dominating odd-carbon atom number compounds is produced.

**Fig. 4:**
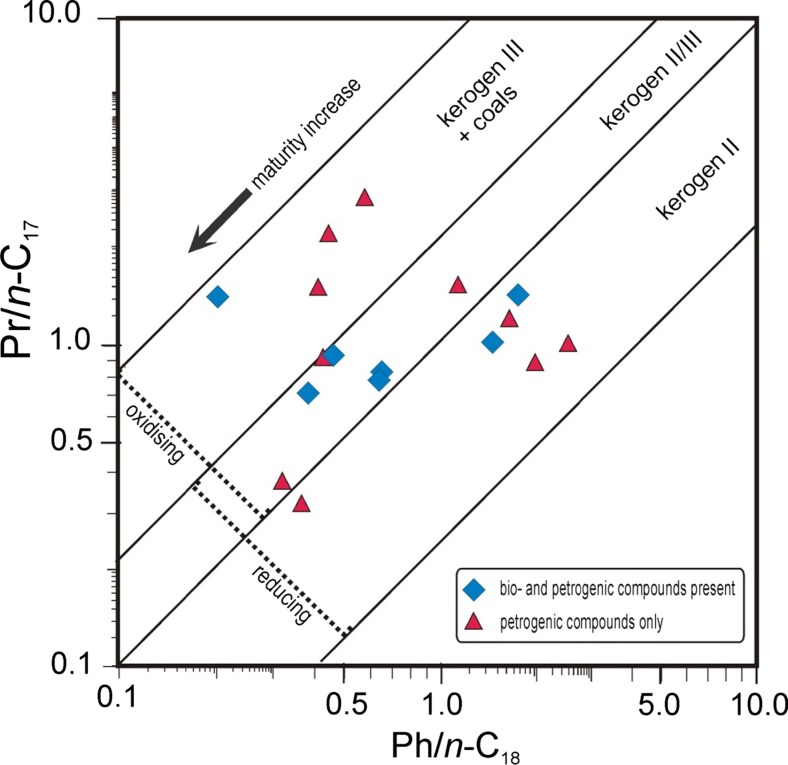
Hunt diagram (Hunt 1996); *Pr—*pristane, *Ph—*phytane

Pristane and phytane belonging to acyclic terpenes are well preserved in DEP and thus it was possible to calculate Pr/Ph, Pr/*n*-C_17_, and Ph/*n*-C_18_ ratios (Table 4). Whereas *n*-alkanes in DEP come from two different sources: biological and geochemical, these compounds are related to fossil fuels only. The Pr/Ph ratio values were in the range from 0.25 (anoxic depositional environment) to 6.31 (oxic environment or kerogen III) (Didyk et al. 1978). The Hunt diagram (Fig. 4) shows that most ratio values indicate oils generated from kerogen II (bacterial/algal) and mixed kerogen II/III (algal/terrestrial) formed in a reducing depositional environment. These types are the commonest occurring oils (Hunt 1996). Only four samples (3-5, and 18) can be ascribed to kerogen III and oxic environment of deposition. These results agree with Pr/Ph values estimating the oxicity of the depositional environment. It seems that despite the influence of the combustion process, values of these geochemical ratios are well preserved.

**Fig. 5:**
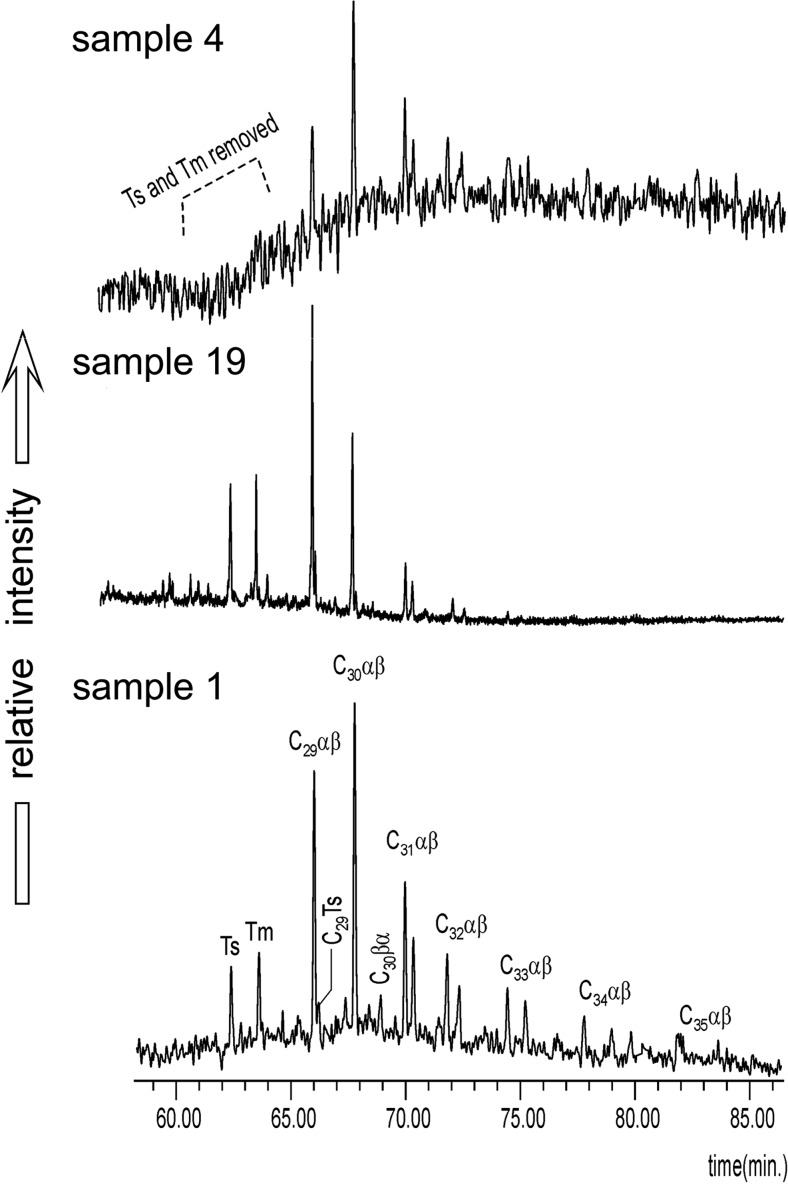
Pentacyclic triterpanes in DEP extracts (*m/z* = 191); samples: a, 4; b, 19; c, 1; abbreviations: *Ts*—18α(H)-22,29,30-trisnorneohopane, *Tm*—17α(H)-22,29,30-trisnorhopane, *C*_*29*_ *αβ*—17α(H),21β(H)-29-norhopane, *C*_*29*_ *Ts*—18α(H)-30-norneohopane, *C*_*30*_ *αβ*—17β(H),21α(H)-29-hopane C_30_, *C*_*30*_ *βα*—17α(H),21β(H)-29-hopane, *C*_*31*_ *αβ*—17α(H),21β(H)-29-homohopanes, *C*_*32*_ *αβ*—17α(H),21β(H)-29-bishomohopanes, *C*_*33*_ *αβ*—17α(H),21β(H)-29-trishomohopanes, *C*_*34*_ *αβ*—17α(H),21β(H)-29-tetrakishomohopanes, *C*_*35*_ *αβ*—17α(H),21β(H)-29-pentakishomohopanes

Pentacyclic triterpanes (hopanes) were found in more than half of the DEP extracts investigated here (Fig. 5). Their distributions range from a well-preserved example of a crude oil type (Fig. 5(c)) comprising compounds from C_27_ (18α(H)-22,29,30-trisnorneohopane, abbreviated as Ts) to C_35_ (17α(H),21β(H)-29-pentakishomohopanes) to distributions strongly affected by the heat of combustion which lost 18α(H)-22,29,30-trisnorneohopane (Ts) and 17α(H)-22,29,30-trisnorhopane (Tm) (Fig. 5(a)). However, the presence of hopanes raises the question of whether they come from diesel fuel itself or from engine lubricating oils, as is sometimes assumed (Sheesley et al. 2009; Huang et al. 2013). Since these compounds’ molecular weight ranges from 370 to 482 Da, oil fractions used for diesel fuel production (b.p. = 250–350 °C) can contain them only in very small amounts. Generally, these compounds occurred in PM in which bioadditives were not preserved, i.e., those which contained only oil-originated compounds (with exception of the 18 sample).

**Fig. 6:**
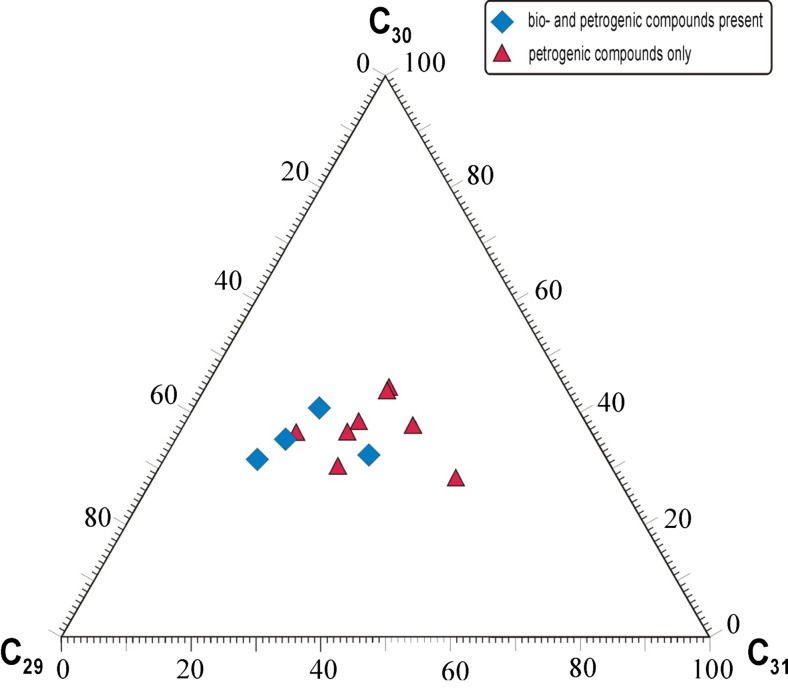
Triangle diagrams showing relative percentage contents of C_29_, C_30_, and C_31_ hopanes in DEP extracts

Values of hopane ratios (Table 4) indicate mature organic matter, being at middle catagenesis at least, which fits well to the fossil fuel range. The relative percentage contents of C_29_, C_30_, and C_31_ hopanes show intermediate variability of distribution, with four samples containing only petrochemical compounds grouped together (Fig. 6).

Steranes (*m/z* = 217) occurred in only a few extracts; only those without preserved bioadditives. Distributions were poorly preserved, with stigmastane diastereomers (C_29_) best seen in the chromatograms, which enabled values of their ratios to be calculated (Table 4). Values of both C_29_αααS/(S+R) and C_29_ααα/(C_29_ααα+C_29_αββ) indicate middle and advanced catagenesis, which agrees with the assessment of maturity based on hopane ratios. Mostly, C_29_ showed equal or higher concentrations than cholestanes (C_27_), except the three sample (C_27_ααα/C_29_ααα <1.0). This may indicate kerogen III-originating crude oils or poorer preservation of lighter steranes (Seifert and Moldowan 1986).

**Fig. 7:**
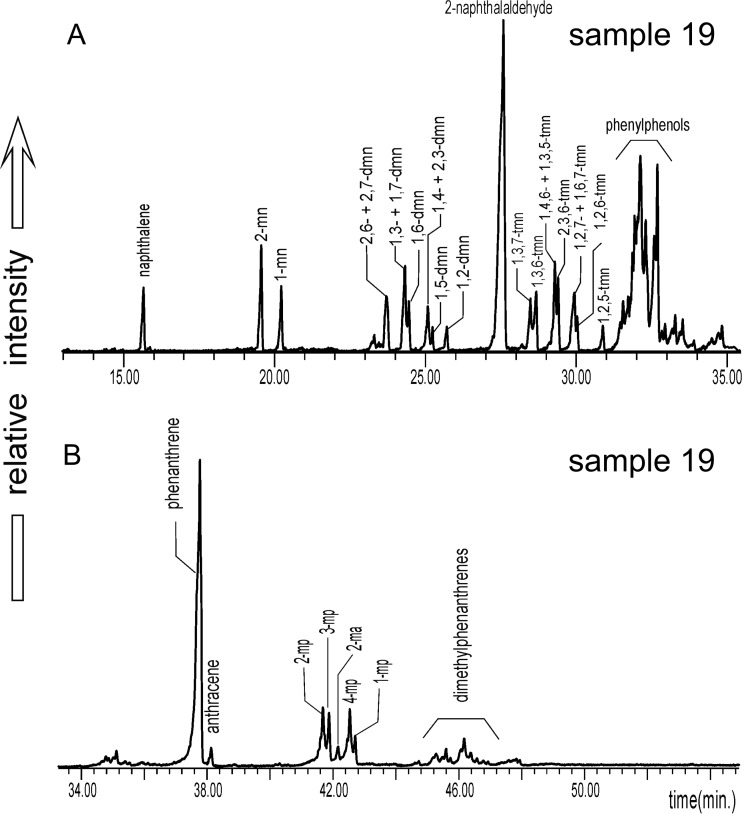
Ion chromatograms of (*a*) alkylnaphthalenes (*m/z* = 128, 142, 156, 170) and (*b*) alkylphenanthrenes (*m/z* = 178, 192, 206) occurring in DEP extracts (sample 19); abbreviations: *mn* = methylnaphthalene, *dmn* = dimethylnaphthalene, *tmn* = trimethylnaphthalene, *ma* = methylanthracene, *mp* = methylphenanthrene

**Fig. 8:**
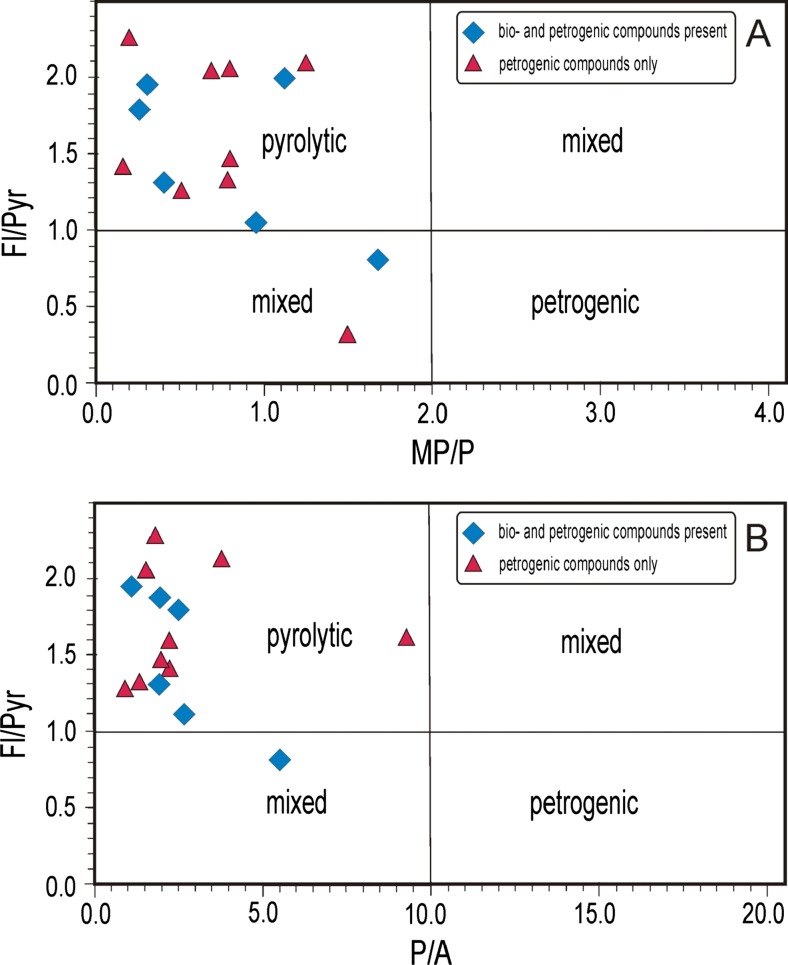
Fluoranthene/pyrene versus methylphenanthrenes/phenanthrene ratios (**a**) and fluoranthene/pyrene versus phenanthrene/anthracene ratios (**b**)

Alkyl aromatic hydrocarbons are applied mostly to assess the thermal maturity of sedimentary organic matter (Radke 1988). The samples investigated contained alkyl phenanthrenes, alkyl pyrenes, and alkyl chrysenes (distributions shown Fig. 7). Alkyl naphthalenes and alkyl biphenyls, common in oils and coals, were mostly absent, possibly due to the influence of combustion; only 15, 17, and 19 samples contained them. Calculated maturity parameters show a wide range of values (Table 5). It seems that combustion particularly strongly affects distributions of lighter alkyl PAHs. The most reliable data come from heavier compounds; however, methylpyrenes and methylchrysenes are not common components of the particulate extracts investigated in this study. Methylphenanthrenes are the most common, and their indices (MPI-1 and MPI-3) give good data. Values of calculated vitrinite reflectance (R_c_, Table 5) correspond to the oil window (Tissot and Welte 1984). Plotted values of the methylphenanthrenes/phenanthrene ratio (MP/P) versus Fl/Pyr in Fig. 8a indicate a predominantly pyrolytic origin (i.e., from combustion) of PAHs in the samples investigated. Only two samples fell into the mixed range. The same pattern occurs for the phenanthrene/anthracene ratio (P/A in Fig. 8b).

The distribution of these substances and the values of the geochemical ratios thereby derived are unequivocally connected to the emission of fossil fuels burned in compression ignition engines. The effect of the exothermic combustion process is limited to compounds of low molecular weight, which shows that the applied methodology permits source identification of PAHs coexisting in the particulate emitted, as well as geochemical markers. The latter compounds show recognizable distributions, typical for fossil fuels, and the values of their ratios fall into ranges corresponding to middle or advanced catagenesis.

Aftertreatment of diesel exhaust gas seems to remove most of geochemical biomarkers and PAHs from the PM, indicating a successful cleaning process. However, bioadditives present in diesel fuel are preserved in PM, in many cases even better than geochemical biomarkers. Thus in the future, investigation of fatty acid profiles may help in distinguishing PM from this source, facilitating future research on atmospheric PM. Particulate matter from the EUDC test cycle contained higher concentrations of organic compounds with more complete distributions of biomarkers, compared to analogous PM from the UDC test cycle.

## Conclusions

The investigations of DEP extracts show that the distributions of geochemical markers, alkyl aromatic hydrocarbons, and polycyclic aromatic hydrocarbons, together with the values of the geochemical ratios thereby derived are unequivocally connected to the emission of fossil fuels burned in compression ignition engines. The effect of the exothermic combustion process is limited to compounds of low molecular weight, which shows that the applied methodology permits source identification of PAHs coexisting in the particulate emitted, as well as geochemical markers. The latter compounds show recognizable distributions, typical for fossil fuels, and the values of their ratios fall into ranges corresponding to middle or advanced catagenesis, the same as fossil fuels combusted.

Aftertreatment of diesel exhaust gas seems to remove most of geochemical biomarkers and PAHs from the PM, indicating a successful cleaning process. However, bioadditives present in diesel fuel are preserved in PM, in many cases even better than geochemical biomarkers. Thus in the future, investigation of fatty acid profiles may help in distinguishing PM from this source, facilitating future research on atmospheric PM. Particulate matter from the EUDC test cycle contained higher concentrations of organic compounds with more complete distributions of biomarkers, compared to analogous PM from the UDC test cycle.
